# The Horizon of Science

**DOI:** 10.7759/cureus.75073

**Published:** 2024-12-03

**Authors:** Bruno Bordoni

**Affiliations:** 1 Physical Medicine and Rehabilitation, Foundation Don Carlo Gnocchi, Milan, ITA

**Keywords:** diaphragm, evidence-based medicine, fascia, impact factor, osteopathic medicine

## Abstract

The core of science is reasoning; assessment tools are an integral part of it. Mathematical/statistical calculation is a tool and is not equivalent to clinical reasoning. Statistics establish mathematical significance but do not always equally highlight clinical relevance. The editorial wants to renew the importance of literature while remembering the true meaning of evidence-based medicine, the real value of the impact factor, and p < 0.05. Dogmas are associated with a particular doctrine, not with the scientific path itself. Science, research, and clinicians should always remember the reason that guides their work, the well-being of the human being.

## Editorial

Science is a constantly evolving body of knowledge: a circle that expands and includes what has been defined as knowledge [1]. In reality, what we define as knowledge, that is, something established, if compared with the dark area of what escapes our understanding, cannot be ascribed as knowledge but as partial knowledge. If it is partial knowledge and does not represent the entirety of what must be learned intellectually, science is probably not a certainty; it is not a point of arrival. Science becomes a set of questions that arise from the awareness of what is missing to fill the completeness of knowledge (if there is ever a boundary). It would be better to say that science is a set of cognitive tools used with the perpetual aim of filling our ignorance.

Science cannot teach, but it can encourage us to know. Science is not dogma, because it is not a religion. Science is not a constraint because it is not a finish line but is always a starting point. It is like a horizon, where there is the illusion of getting closer, but you never really get to touch it.

A current limit to science is the routine lack of collaboration in research by different figures. The goal becomes publishing and not transmitting. This does not allow for growth in knowledge, but a deepening of what we know [1].

**Figure 1:**
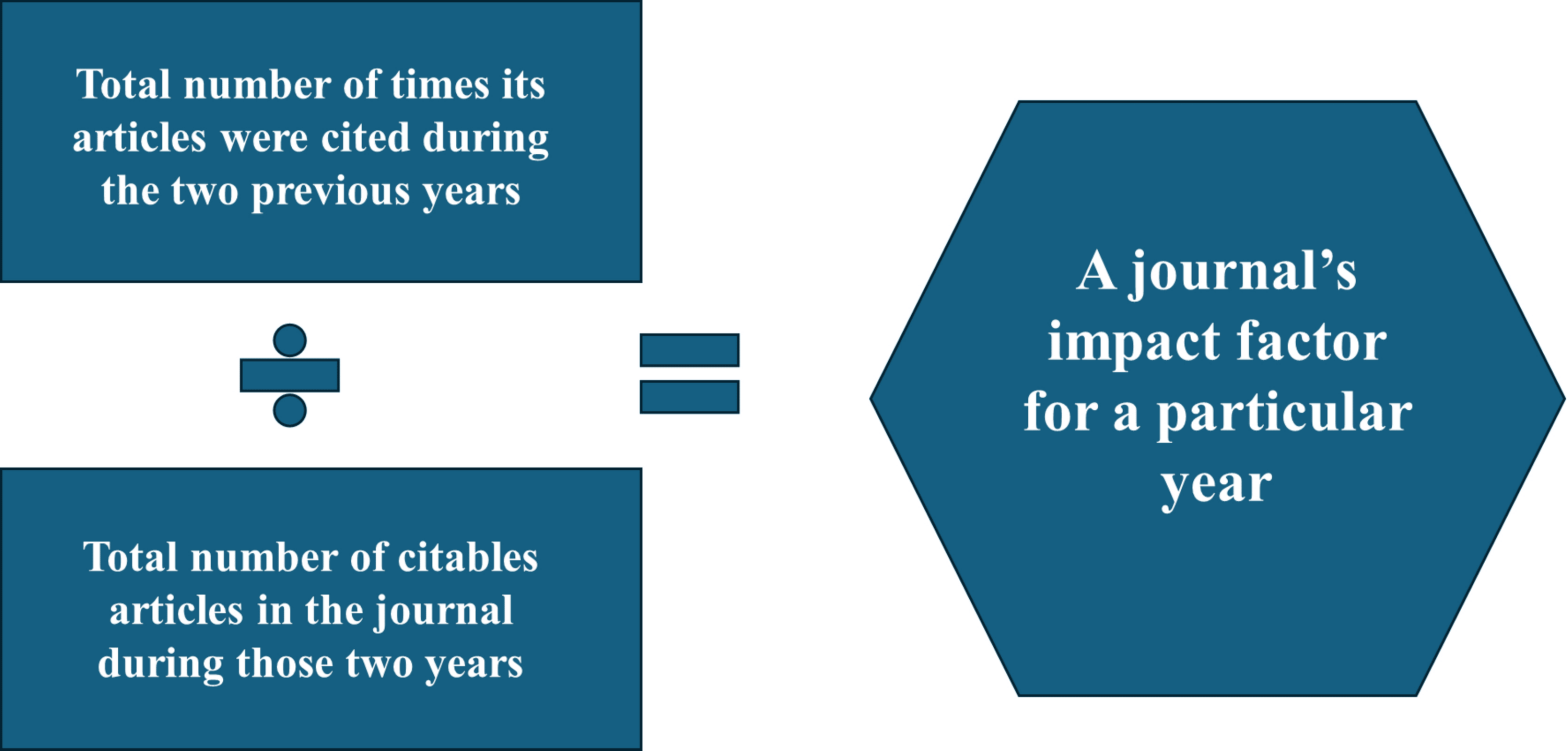
The figure illustrates the mathematical calculation of the impact factor (IF). Divide the number of citations received in the year (numerator) in which you want to calculate the IF, using the number of articles published in the previous two years, by the total number of citable articles published (denominator) by the same journal in the previous two years. To give an example, let's calculate the hypothetical IF of a journal for the year 2022. Numerator = the citations received in 2022 by articles published in 2020 and 2021. Hypotheses 120 and 135, respectively. Denominator = number of articles published in 2020 and 2021. Hypotheses 80 and 95, respectively. So we will have: IF 2022 = (120+135)/(80+95) = 255/175 = 1.5

We look at the so-called “impact factor” (IF) of a journal, as science becomes a market. The idea of IF was first proposed by Eugene Garfield, PhD, in 1955; in 1961 Garfield and Dr. Irving H. Sher published the meaning of IF with the Institute for Scientific Information [2]. The IF is a bibliometric index, previously owned by the publisher Thomson Reuters and later (2016) sold to private equity firms Onex Corporation (Canadian investment management company, 1984) and Baring Private Equity Asia (financial services). The sale of the brand led to the creation of the company Clarivate Analytics., from which IF currently derives. The Clarivate (which actually trades on the New York Stock Exchange) calculates the IF of its journals, which are then published in Journal Citation Reports. IF measures the average number of citations received, in the reference year considered, by articles published by a scientific journal in the two previous years; it is therefore an indicator of the performance of scientific periodicals, which expresses the impact of a publication on the scientific community of reference. IF does not express a value in itself; it is not connected to the presence of a peer review process nor to the quality of the published articles [3]. IF measures the average of how many times the same article (citation) appears in a period of time. It is a mathematical calculation. The journal that gets more citations will get a higher IF. IF is a number that reflects the ability of a journal to publish articles that will probably have a high number of citations; IF is a number that varies over time [2]. IF does not reflect the value of the article or the content of the journal [3]. According to Garfield, the same journal can present different quality articles, despite the same IF of the journal [2,3]. This mechanism puts the value of researchers on the same level, regardless of the topic and the novelty/content of the text [2,3]. This purely mathematical mechanism starts only three years after the first publication, and once again, only for non-qualitative mathematical rules of the content of the research, an inequality between journals is created (Figure 1) [3].

Another question that seems to be connected to the mathematical logic of IF is whether evidence-based medicine (EBM) reflects this calculation. The concept of EBM was first encountered in 1991 in the journal of the American College of Physicians, through an editorial written by Gordon Herry Guyatt, Dr., who was influenced by his professor David Sackett, Dr. [4]. The International EBM Working Group was then created, with the aim of putting the idea described into practice, publishing a guide to help correctly read the meaning of scientific research [4]. Guyatt's proposal was based on implementing and enriching the doctor's experience with the knowledge acquired in literature, as the clinician must remain updated and not rely solely on his own experiential ability. Sackett wrote an article in 1996 to recall the conceptual foundations on which EBM is based, probably to try to contain the extremist drift of the 1991 idea, for which several clinicians began to use this assumption as an absolute constraint [4].

EBM was born as a philosophical current and pushes to use the knowledge acquired from research and literature to have a "carnet" of broader choices for the therapeutic decision, always respecting the patient's subjectivity. EBM means trying to replace clinical approaches that are considered obsolete with other diagnostic and practical strategies that are more correct [5].

This does not mean that only what is proven or published on PubMed is valid, while what is not proven (or does not yet exist) is a clinical error. We would not have aspirin or other extraordinary innovations without the innovative idea or a starting hypothesis. Experimental studies are useful to begin to ascertain whether the ideas or hypotheses are effectively valid, just as review articles are important, both systematic and narrative, as they provide further ideas and/or indications on how to continue the research. By accepting EBM as an axiom, the clinician could not follow any pharmacological path that is not visible in the literature, distancing the patient. As Sackett himself writes: “Good doctors use both individual clinical expertise and the best available external evidence, and neither alone is enough” [5].

**Figure 2:**
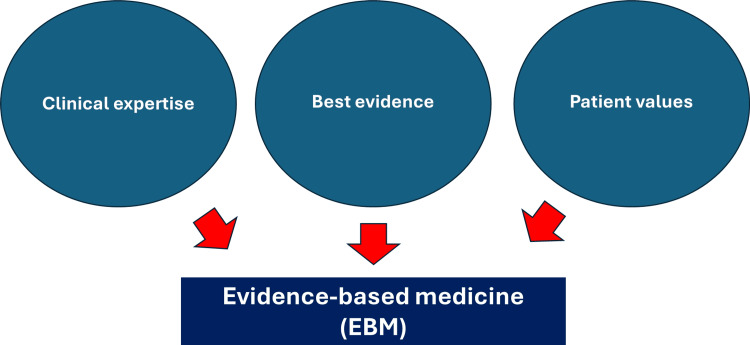
The figure highlights the equal importance of the clinician's experience, the patient's value, and clinical data from the literature: this triptych forms evidence-based medicine (EBM).

EBM is not an insurmountable limit but a point of reference around which the subjectivity of the clinician and the patient always revolves. If the compass indicates direction, regardless of the tortuosity of the road, we will be the ones to choose which path to follow with respect to the pre-established goal. Clinical experience should not be “tyrannized” by current research, as knowledge is always partial [5]. Sackett writes: “External clinical evidence can inform, but can never replace, individual clinical expertise, and it is this expertise that decides whether the external evidence applies to the individual patient at all and, if so, how it should be integrated into a clinical decision” [5]. Using the acronym EBM to remove the value of subjective clinical experience, as well as the subjective experience of care by the patient, is like putting up sterile conceptual “barricades” (Figure 2) [5].

Experimental research, to be consistent, non-confusing, and publishable, must follow some dictates attributable to statistics. It follows that, often, groups of patients are divided (chosen) based on some characteristics and not based on their real needs. The results, which must start from the reproducibility of the data (classification of the patient by choice of the type of study and the objective of the research), will be tied to the organization of the study carried out. Theoretically, this should not happen. In fact, many risk-of-bias scales reduce the quality scores of these types of papers. Ideally, clinical randomized controlled trials (RCTs) should start from two or more arms with similar patients (characteristics). But it is precisely this characteristic of choosing similar patients that removes from the statistical calculation other patients with similar pathology but with additional characteristics that would alter the mathematical result. Clinically "similar" and "stable" patients are chosen and only with certain characteristics because otherwise, the results will be confusing and "contaminated" by non-reproducible factors or clinical causes (real factors), which factors/clinical causes are omitted from the statistical calculation of the data derived from the research. This means that the responses of trials are limited and constrained by the main objective of the study itself [4]. The best available evidence does not necessarily represent the only or the real possible therapeutic choice for each patient [4,5]. Clinical dogma is an impediment to the growth of knowledge, which is always partial. All classifications that derive from EBM, such as evidence-based practice (EBP), which is further based on patient subjectivity, must be a clinical aid and not an imposition. EBM and EBP are not schemes in which the clinician is a prisoner; rather, they are models of reasoning whose center is always the patient.

A numerical value is used to determine the effectiveness of a clinical strategy compared to another, that is, the p < 0.05 (set of collected data) [4]. Normally in inferential statistics, a p-value greater than 0.05 indicates that the hypothesis sampled is null. Vice versa, a p-value less than 0.05 suggests that the hypothesis of the observed data is statistically significant. To calculate the p-value, a series of different mathematical methods can be used, based on the need and the type of group that the research wishes to evaluate. P-values are influenced by some different factors: the size of the sample, the size of the effect, and the type of test with its hypotheses. The larger (sample size) the group, the faster you will get statistically significant results with small differences and vice versa. The larger the effect size, the faster you will get statistically significant results, even with smaller groups, and vice versa. A test becomes more sensitive to differences under certain assumptions, such as the distribution of the data, independence of measurements, homoscedasticity (random variables), one-sided or two-sided, and between groups or within a group. A huge study can find statistically significant results even with small effects. These effects may not have any clinical significance. The original penicillin study used a tiny sample to show that the effects on killing bacteria are huge. The p-value indicates only the probability of obtaining the observed result or a more extreme result under the null hypothesis. It does not provide information about the size or clinical significance of the effect or association. A non-significant p-value only suggests that the observed result is not statistically significant but does not necessarily mean that there is no effect or association. It may be due to low statistical power or other factors, such as measurement error or confounding variables. Furthermore, p-values in systematic reviews may hide errors of interpretation, as the homogeneity of inclusion/exclusion criteria is not always considered, with a result that is not necessarily a true value [4].

The core of science is reasoning; assessment tools are an integral part of it. Mathematical/statistical calculation is a tool and is not equivalent to clinical reasoning. Statistics establish mathematical significance but do not always equally highlight clinical significance.

In conclusion, the ultra-specialization of a single scientific field must not obscure the birth of new disciplines (for example, quantum biology) or base the understanding of the human body only on its own point of view. The body includes all scientific disciplines. Every small or large gear of a clock has equal value for the final functioning. Every subject of study that involves the patient's health is necessary for clinical and therapeutic completeness. And precisely because of the explosive amount of data in the literature that emerges from PubMed, the increasingly precise evaluation from the macroscopic to the nanoscopic, we must not ignore clinical behavior towards the single biological entity: the human being.
